# Value of combining PET/CT and clinicopathological features in predicting EGFR mutation in Lung Adenocarcinoma with Bone Metastasis

**DOI:** 10.7150/jca.46414

**Published:** 2020-07-11

**Authors:** Guangyu Yao, Yiyi Zhou, Yifeng Gu, Zhiyu Wang, Mengdi Yang, Jing Sun, Quanyong Luo, Hui Zhao

**Affiliations:** 1 Department of Internal Oncology, Shanghai Jiao Tong University Affiliated Sixth People's Hospital, Shanghai, 200030, China.; 2 Department of Radiology, Shanghai Jiao Tong University Affiliated Sixth People's Hospital, Shanghai, 200030, China.; 3 Department of Nuclear Medicine, Shanghai Jiao Tong University Affiliated Sixth People's Hospital, Shanghai, 200030, China.

**Keywords:** Adenocarcinoma, Bone metastasis, EGFR mutations, PET/CT, SUVmax

## Abstract

**Purpose:** Epidermal growth factor receptor (EGFR) mutation is the most common target for precision treatment in metastatic lung adenocarcinoma. We investigated the predictive role of ^18^F-FDG PET/CT and clinicopathological features for EGFR mutations in lung adenocarcinoma with bone metastasis.

**Methods:** Seventy-five lung adenocarcinoma patients with histologically confirmed bone metastasis were included. They all received EGFR status test and PET/CT before systemic treatment. The differences of maximum standardized uptake value (SUVmax) in primary tumor (pSUVmax), regional lymph node (nSUVmax) and bone metastasis (bmSUVmax) between different EGFR status groups were compared, alongside with common clinicopathological features. Multivariate logistic regression analysis was performed to evaluate predictors of EGFR mutations.

**Results:** EGFR mutations were found in 37 patients (49.3%). EGFR mutations were more common in females, non-smokers, expression of Thyroid Transcription Factor-1 (TTF-1) and NaspinA. Low bmSUVmax was significantly associated with EGFR mutations, while no significant difference was observed in pSUVmax and nSUVmax. Multivariate analysis showed that bmSUVmax ≤7, non-smoking, expression of TTF-1 were predictors of EGFR mutations. The area under the curve (AUC) of receiver operating characteristic (ROC) curve was 0.84 for the combination of the three factors.

**Conclusion:** Low bmSUVmax is more frequently in EGFR mutations, and bmSUVmax is an independent predictor of EGFR mutations. Combining bmSUVmax with other clinicopathological features could forecast the EGFR status in lung adenocarcinoma with unavailable EGFR gene testing.

## Introduction

Precision medicine has become a prevailing approach in the treatment of malignant tumors 1. A typical representative is the EGFR mutations 2, which is the most common druggable target in non-small cell lung cancer (NSCLC) 3. In metastatic NSCLC with EGFR mutations, tyrosine kinase inhibitors (TKI) have been established in front-line treatment status 4. Now the molecular profiling of EGFR status in advanced NSCLC is a recommended standard 5, 6. However, in clinical practice, a successful gene analysis depends on sufficient tumor samples of good quality, which can be sometimes difficult to obtain 7, 8. Even in some clinical trials, effective detection rate of EGFR was no more than 40% 4, 9. Furthermore, in a real-world study, the proportions of patients receiving TKI therapy among those with unknown EGFR mutation status could be as high as 45.8% 10. Seeking some other predictors of EGFR status may offer assistance.

^18^F-FDG PET/CT is a noninvasive diagnostic and staging tool for NSCLC 11. FDG uptake was associated with tumor invasiveness 12, meanwhile EGFR signaling regulated cell proliferation and glucose metabolic pathway in NSCLC with EGFR mutations 13. Using FDG tumor uptake as a predictor of EGFR status could be worth exploring. Several studies have investigated the association between SUVmax and EGFR mutations 8, 14-25. However, the results were controversial and conflicting. Previous studies included NSCLC patients with I-IV stages and various histological subtypes, which increased diversity. Furthermore, most studies focused on the SUVmax of primary lung tumor, only few of them 8, 14 discussed the relationship between metastatic FDG uptake and EGFR status. EGFR status in NSCLC may vary according to the tumor staging or histological subtypes, and heterogeneity could exist between primary and metastatic site, making results from above researches inconclusive. Therefore, in this retrospective study, we aimed to evaluate whether ^18^F-FDG PET/CT and common clinicopathological features could predict EGFR status in advanced lung adenocarcinoma with histologically confirmed bone metastasis.

## Materials and Methods

### Patients

This retrospective study included lung adenocarcinoma patients with histologically confirmed bone metastasis from November 2016 to November 2019. The study was approved by the ethics committee of our hospital and formal consent was waived. An initial 80 patients were included according to inclusion criteria: PET/CT scan indicated suspicious bone metastasis; bone biopsy confirmed metastasis from lung adenocarcinoma; available results for EGFR mutation status. Then 5 patients were excluded due to exclusion criteria: treatment before PET/CT within 6 months (2 patients); interval between PET/CT and bone biopsy exceeded 1 month (3 patients). Finally, 75 patients were included.

### ^18^F-FDG PET/CT

**Figure 1:**
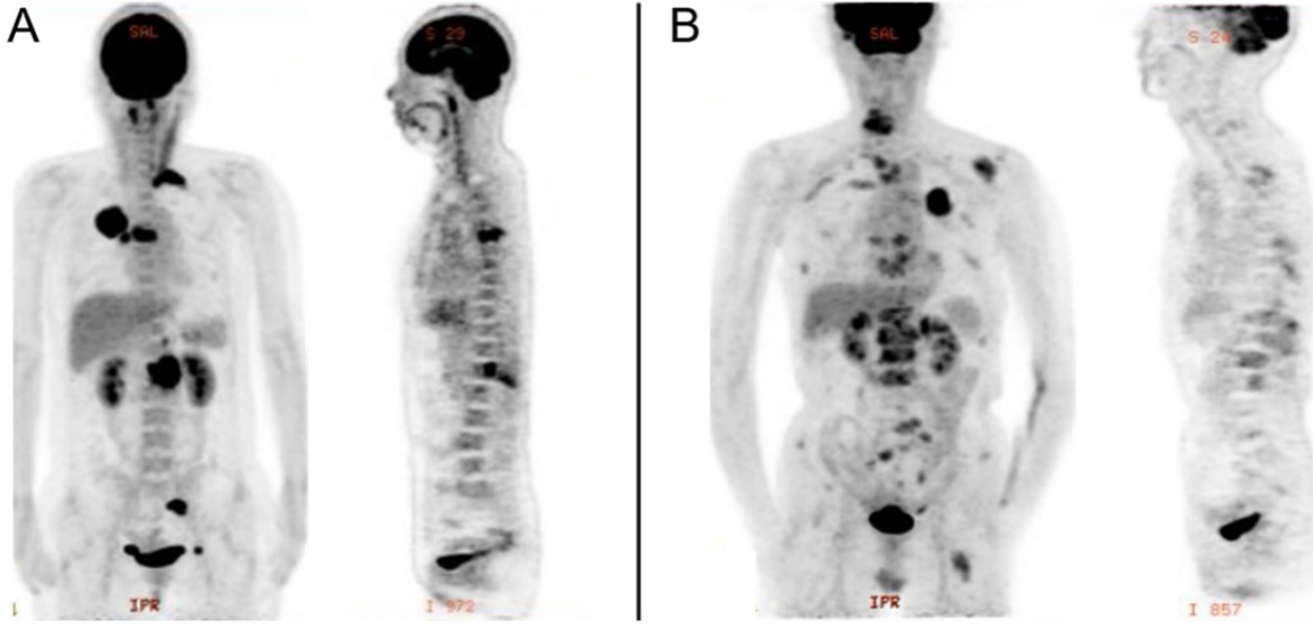
PET image of stage IV lung adenocarcinoma with bone metastasis. A, EGFR-wild: primary tumor in the right upper lobe (SUVmax, 20.3), with regional node (SUVmax, 11.4), and bone metastasis (SUVmax, 16.0). B, EGFR-mutant: primary tumor in the left upper lobe (SUVmax, 9.6), with intrapulmonary (SUVmax, 2.6), distant node (SUVmax, 2.4), adrenal gland (SUVmax, 6.1), and bone metastasis (SUVmax, 6.0).

PET/CT was performed using an integrated PET/CT system (Discovery VCT; GE Medical Systems). All patients were required to fast for at least 6h and undergo a peripheral blood sugar test to avoid hyperglycemia. Approximately 1 h after the intravenous injection of ^18^F-FDG (3.7MBq/kg), CT was done from head to lower limbs with the following setting: 120 V and 80 mA, 64 slices, with a slice thickness of 3.75 mm. PET scans were performed with 2.5 min per bed position. Finally, the CT and PET images were reconstructed iteratively using ordered subset expectation maximization. Attenuation correction was done by unenhanced CT. A senior nuclear medicine doctor then evaluated all of the combined ^18^F-FDG PET/CT scans whilst blinded to the EGFR status. The region of interest (ROI) over the primary lung tumor, regional nodal metastasis and bone metastasis were drawn on PET/CT images on each transaxial slice. SUVmax was defined at the peak value on one pixel with the highest counts within the ROI. Representative image is shown in Figure 1.

### Pathological evaluation and EGFR mutation

Bone biopsy was performed in each patient. The specimen was then handled with modified Ethylene Diamine Tetraacetic Acid (EDTA) decalcification (which is good at preserving antigenicity and DNA quality), instead of traditional acid decalcification. If bone metastasis form NSCLC was confirmed by the morphology and immunohistochemistry (IHC) results of specimen, EGFR mutations were analyzed under of the amplification refractory mutation system (ARMS) using the EGFR 29 Mutations Detection Kit (Amoy Diagnostics, Xiamen, PRC).

### Statistical analysis

The characteristics of included patients were compared, using Fisher's exact test for binary data and the Wilcoxon rank-sum test for continuous data. All tests were two-sided and P values less than 0.05 were considered statistically significant. ROC curve was constructed to obtain the cutoff value of bmSUVmax in predicting EGFR mutation status. Logistic regression analysis was performed to identify independent predictors of the EGFR status. Variables with p < 0.05 in the multivariate analysis were independent predictors for EGFR mutations. The ROC curves were constructed for individual predictor and combined factors in predicting EGFR mutations. Statistical analyses were performed using STATA/SE version 15.1 (StataCorp LLC USA).

## Results

### Patient and clinical characteristics

Among the final 75 patients, all of them received EGFR status test from bone biopsy. 66 patients received PET/CT as an initial diagnosis. 9 patients had resection of primary site before PET/CT, and then PET/CT was performed as an overall assessment (The interval between resection and PET/CT was not exceed 1.5 months). EGFR mutations were identified in 37 (49.3%). The mutations types were Exon 18 (2, 5.5%), Exon 19 (22, 59.5%), Exon 21 (13, 35%). Further clinical characteristics were summarized in Table 1.

### SUVmax of tumors

**Figure 2:**
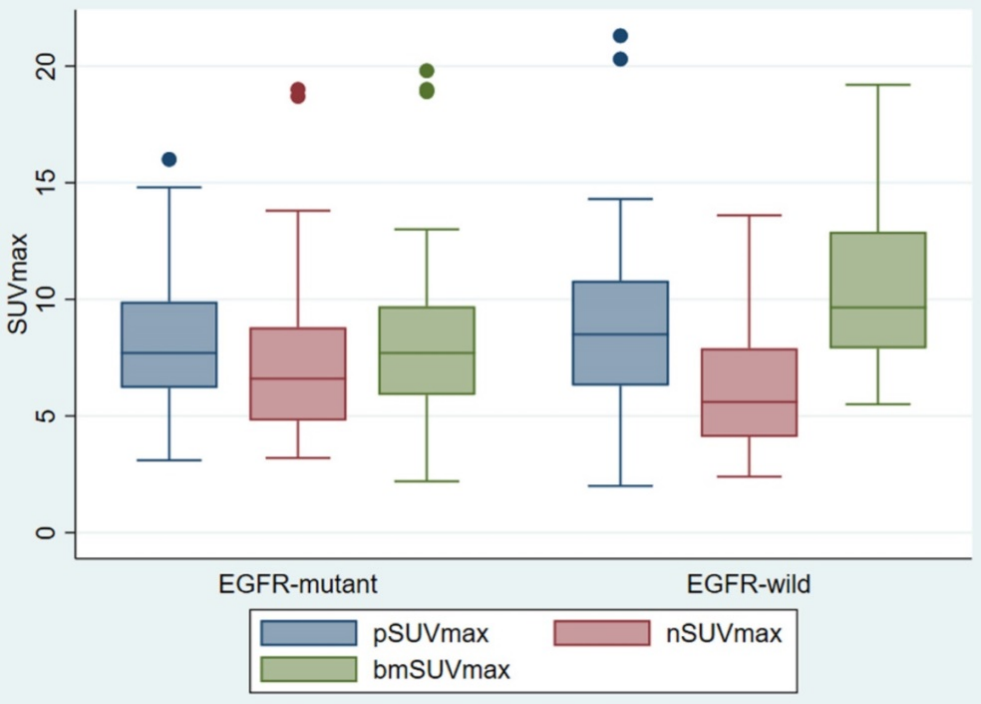
Box Plot of SUVmax of primary tumor, regional lymph node, and bone metastasis, according to different EGFR status.

pSUVmax, nSUVmax and bmSUVmax were compared according to the EGFR status (Figure 2). There was no difference in the pSUVmax (median 7.7 vs 8.5, p < 0.454) and nSUVmax (6.6 vs 5.6, p < 0.208) between the EGFR mutant and EGFR wild groups. The EGFR mutant bone metastasis had lower bmSUVmax (median 7.7 vs 9.7, p < 0.015). The ROC curve showed the cutoff value of bmSUVmax was 7.0, with sensitivity 45.9%, specificity 84.2% and AUC 0.65 (p < 0.01).

### Prediction of the EGFR mutation status

**Figure 3:**
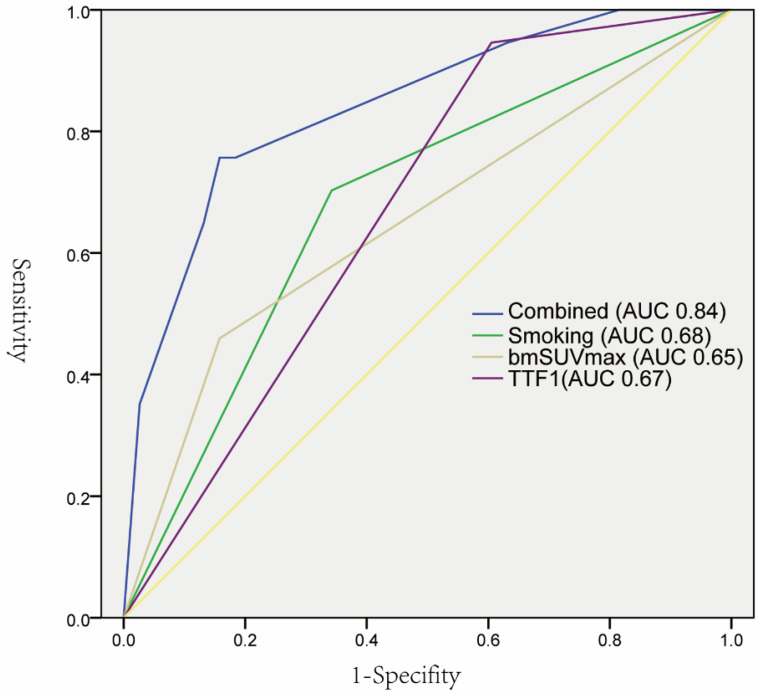
ROC curves of individual predictors and combined factors (bmSUVmax, smoking status, and TTF-1) in predicting EGFR mutation status.

As summarized in Table 1, EGFR mutations were found more frequently in female patients (21 vs 12, p 0.037), non-smoking (66.67% vs 33.33%, p 0.003), lower bmSUVmax (7.7 vs 9.7, p 0.015), positive expression of TTF-1(60.34% vs 39.26%, p 0.001), and NaspinA (59.65% vs 40.35%, p 0.002). The univariate logistic regression analysis showed that five factors were significantly correlated with EGFR mutations. The subsequent multivariate regression analysis demonstrated that non-smoking, lower bmSUVmax, and positive expression of TTF-1 were independent predictors of EGFR mutations (Table 2). The ROC curves analysis revealed that each factor could predict EGFR mutation with AUC ranging from 0.65 to 0.68. When the three factors were combined, the AUC could reach 0.84 (Figure 3).

## Discussion

EGFR mutations had a decisive role in systematic therapy of NSCLC. In this study, we found that bone metastasis from EGFR mutant lung adenocarcinoma had lower SUVmax than EGFR wild types. However, SUVmax of primary tumors and regional lymph nodes didn't seem significantly different between two EGFR status lung adenocarcinomas. Further analyses demonstrated that lower bmSUVmax was an independent predictor for EGFR mutations. When combining with other accessible factors, an AUC of predicting EGFR mutations could reach 0.84.

Several studies 8, 12-23 had evaluated the value of FDG uptake for predicting EGFR status in NSCLC. As described in Table 3, the results were not very consistent. Almost all previous studies solely focused on the pSUVmax. Ten of them 8, 14-16, 18-23 revealed that lower pSUVmax was associated with EGFR mutations, and three studies 8, 22, 23 found lower pSUVmax was an independent predictor for EGFR mutations by multivariate analysis. Meanwhile, two studies 15, 19 demonstrated that high pSUVmax was a significant predictor of EGFR mutations, and one 14 didn't show statistical difference in pSUVmax between different EGFR statuses. Possible explanation for these controversies may the clinicopathological features. These studies included NSCLC patients of all stages and various histological types. EGFR status could vary according to the different clinical stage 8, while NSCLC FDG uptake across histologic subtypes may be discrepant 26. As a result, conflicts existed in the previous related studies.

Our included patients contained only stage IV lung adenocarcinoma with bone metastasis. Final results demonstrated lower bmSUVmax was significant predictor of EGFR mutations, while pSUVmax and nSUVmax weren't statistically different. Few studies identified predictive values of FDG uptake in primary tumor, lymph node, and metastasis simultaneously. Lee et al. 14 reported 71 stage IV lung adenocarcinoma patients, finding SUVmax of metastasis (both nodal and distant) was a significant independent predictor of EGFR mutations, meanwhile SUVmax of primary tumor wasn't significant. Another study by Lv et al. 8 included 849 NSCLC patients with different stages and histologic subtypes. In their analyses, low SUVmax of primary tumor, lymph node, and distant metastasis were associated with EGFR mutations. Furthermore, in a subgroup analysis of stage IV adenocarcinoma, the pSUVmax wasn't meaningful between different EGFR status.

Our data were consistent with above studies to a great extent, whereby low SUVmax of metastasis could predict EGFR mutations of NSCLC, while SUVmax of primary tumor was less useful. EGFR was responsible for the tumor invasiveness, which is an interesting finding that EGFR mutations were associated with lower SUVmax. FDG uptake in malignant tumors was mediated by Glucose transportase-1 (GLUT-1) 27, Higashi et al. 28 proposed the correlations between GLUT-1 expression and FDG uptake in NSCLC. EGFR, on the other hand, has a strong interaction with sodium/glucose cotransporter 1 (SGLT1) in various tumors 29-31. As SGLT1 opposed to GLUT-1 27, this mechanism may partly explaine less FDG avid in EGFR mutations. However, further mechanism studies should be sponsored for investigating the relationship between tumor metabolic activity and EGFR mutations.

In patients with stage IV NSCLC, SUVmax of primary tumor seemed to have no predictive value, a reasonable explanation being heterogeneity in tumor metastasis. Lymph node FDG uptake was also significant in the studies of Lv et al. 8 and Lee et al. 14, nevertheless, our data didn't find significance in different EGFR status. Only 47 patients in the study had regional node involvement, which may affect the statistical analysis. We also considered the confounding factor of inflammatory lymph nodes which is difficult to identify from metastatic nodes.

One novel aspect of our research was the histologic tissue origin. All of the included patients received bone biopsy; subsequent pathological evaluation and EGFR analysis were performed by bone metastasis tissue. Hardness was the distinguishing feature of bone tissue; decalcification should be performed first for histological analyses 32. Traditional acid decalcification inevitably affected IHC 32 and molecular pathology 33. In our study, EDTA decalcification was applied, which was more suitable for preserving the bone structure, antigenicity, and DNA quality 32-34. EGFR mutations were identified in 37 (49.3%), which was consistent with the mutation rate in Asian populations (36.8-76.2%) 35. To our knowledge, our study was the first using bone metastasis tissue as reference, comparing to the similar others. The molecular analysis from same tissue source could avoid metastatic tumor heterogeneity to a great extent.

In the diagnosis of lung cancer, lung biopsy was the more traditional selection for pathological examination and gene test in the past. However, pneumothorax and air embolism arising from lung biopsy could be potentially life-threatening 36, 37. By contrast, bone biopsy was proven to be a safe method without serious biopsy-related complications 38-40. In our clinical practice, biopsy was usually performed by outpatient. Patients left the hospital directly after the biopsy. Consequently, quite a proportion of patients preferred to a safer biopsy method after consideration. As a result, in this study, there existed patients with bone biopsy, rather than lung biopsy.

In the multivariate analysis, we demonstrated another two predictors of EGFR mutations: non-smoking, and positive expression of TTF-1. The predictive value of non-smoking has been proven by previous studies 8, 41. TTF-1, a routine IHC index of lung cancer, may be a biomarker to predict the unknown EGFR mutation status. In a meta-analysis containing 9764 patients 42, TTF-1 expression significantly correlated with EGFR mutations in patients with NSCLC. When specimens weren't good enough for gene testing, those clinicopathological features may assist to guide targeted therapy.

There were several limitations in our study. First, it was a retrospective study that may introduce selection bias. We included lung adenocarcinoma patients with histologically confirmed bone metastasis, further validation of our results would be performed by other stage IV lung cancer. Moreover, not every patient received ALK and ROS1 rearrangement tests; we couldn't know whether other mutations have an influence on FDG uptake. As clinically impractical, not all bone metastasis lesions were biopsied, we chose the bone lesion with highest bmSUVmax to analyze.

In conclusion, the bone metastasis of EGFR-mutant lung carcinoma has lower FDG uptake compared with EGFR wild-type, and SUVmax could be a valuable noninvasive predictor for EGFR mutations. Combining SUVmax with other clinicopathological features could forecast the EGFR status in lung adenocarcinoma with unavailable EGFR gene testing.

## Figures and Tables

**Table 1:** Clinicopathological features and PET/CT parameters in study

Characteristics	EGFR Mutant	EGFR Wild	Total	p value
**Age (years, median)**	63	64.5	64 (37-85)	0.767
**Gender**				
Male/Female	16/21	26/12	42/33	0.037
Non-smoking	26 (66.67%)	13 (33.33%)	39 (52%)	0.003
Primary tumor size (cm, median)	3.8	3.2	3.5	0.979
**Regional node involvement**	25 (53.19%)	22 (46.81%)	47 (63%)	0.476
**Bone metastasis**				
Oligo (<5)	12 (46.15%)	14 (53.85%)	26 (35%)	0.809
Multiple (≥5)	25 (51.02%)	24 (48.98%)	49 (65%)	
Other distant organ metastasis	8 (42.11%)	11 (57.89%)	19 (25.33%)	0.597
**Interval between bone biopsy and PET/CT (day, median)**	6	4	5	0.175
**pSUVmax (median)**	7.7	8.5	8.3	0.454
**nSUVmax (median)**	6.6	5.6	6.4	0.208
**bmSUVmax (median)**	7.7	9.7	8.9	0.015
**CK7**			75 (100%)	
Positive	37 (50.68%)	36 (49.32%)		0.493
Negative	0	2 (100%)		
**Vilin**			72 (96%)	
Positive	11 (42.31%)	15 (57.69%)		0.469
Negative	24 (52.17%)	22 (47.83%)		
**TTF-1**			75 (100%)	
Positive	35 (60.34%)	23 (39.26%)		0.001
Negative	2 (11.76%)	15 (88.24%)		
**NaspinA**			75 (100%)	
Positive	34 (59.65%)	23 (40.35%)		0.002
Negative	3 (16.67%)	15 (83.33%)		
**Ki67**			55 (73%)	
>20%	12 (50%)	12 (50%)		0.789
≤20%	14 (45.16%)	17 (54.84%)		

**Table 2:** Univariate and multivariate analysis of predictors in EGFR status

Characteristics	Univariate Analysis OR (95% CI)	p value	Multivariate Analysis OR (95% CI)	p value
**Gender**				
Female	2.84 (1.11-7.31)	0.030	0.97 (0.17-5.53)	0.971
Male	Reference		Reference	
**Smoking status**				
Non-smoking	4.55 (1.72-12.02)	0.002	6.42 (1.17-35.28)	0.032
Smoking	Reference		Reference	
**bmSUVmax**				
≤7	4.53 (1.53-13.42)	0.006	4.23 (1.02-17.49)	0.047
>7	Reference		Reference	
**TTF-1**				
Positive	11.41 (2.38-54.66)	0.002	11.65 (1.69-80.21)	0.013
Negative	Reference		Reference	
**NaspinA**				
Positive	7.39 (1.92-28.45)	0.004	2.45 (0.43-13.88)	0.311
Negative	Reference		Reference	

**Table 3:** Similar studies of relationship between SUVmax and EGFR mutation status in NSCLC

Author	N	Histology	Stage	EGFR mutation	Lesions Measured	Favor factor in mutation
Lee et al. 11	71	ADC	IV	48 (68%)	pSUVmax; nSUVmax; SUVmax	Low nSUVmax; mSUVmax
Huang et al. 12	77	ADC	III-IV	49 (64%)	pSUVmax	High pSUVmax
Qiang et al. 13	97	ADC	I-III	44 (45%)	pSUVmax	Low pSUVmax
Li et al. 14	115	ADC,	I-IV	64 (56%)	pSUVmax	Low pSUVmax
Minamimoto et al. 15	131	ADC	I-IV	32 (24%)	pSUVmax	Low pSUVmax
Ko et al. 16	132	ADC	I-IV	69 (52%)	pSUVmax	High pSUVmax
Zhu et al. 17	139	ADC	I-IV	74 (53%)	pSUVmax	Low pSUVmax
Yang et al. 18	200	ADC	I-IV	115 (58%)	pSUVmax	Low pSUVmax
Gu et al. 19	210	ADC, non-ADC	I-IV	70 (33%)	pSUVmax	Low pSUVmax
Guan et al. 20	316	ADC, non-ADC	I-IV	126 (40%)	pSUVmax	Low pSUVmax
Yip et al. 21	348	ADC, non-ADC	I-IV	44 (13%)	pSUVmax	Low pSUVmax
Kazuya et al. 22	734	ADC	I-IV	334 (46%)	pSUVmax	Low pSUVmax
Lv et al. 8	849	ADC, non-ADC	I-IV	371 (46%)	pSUVmax; nSUVmax; mSUVmax	Low pSUVmax; nSUVmax; mSUVmax

ADC: adenocarcinoma; pSUVmax: SUVmax in primary tumor; nSUVmax: SUVmax in regional lymph node; mSUVmax: SUVmax in distant metastasis.
